# Glycemic response to acute high‐intensity interval versus moderate‐intensity continuous exercise during pregnancy

**DOI:** 10.14814/phy2.15454

**Published:** 2022-09-19

**Authors:** Jenna B. Wowdzia, Tom J. Hazell, Margie H. Davenport

**Affiliations:** ^1^ Program for Pregnancy and Postpartum Health, Faculty of Kinesiology, Sports and Recreation, Women and Children's Health Research Institute, Alberta Diabetes Institute University of Alberta Edmonton Alberta Canada; ^2^ Department of Kinesiology and Physical Education, Faculty of Science Wilfrid Laurier University Waterloo Ontario Canada

**Keywords:** exercise, glucose, hypoglycemia, pregnant, prenatal

## Abstract

The present study investigated the glycemic response to an acute high‐intensity interval training (HIIT) session (10 one‐minute intervals ≥90% HR_max_ interspersed with one‐minute of active recovery) versus a moderate‐intensity continuous training (MICT) session (30 min at 64%–76% HR_max_) during pregnancy. Twenty‐four normoglycemic females with a singleton pregnancy (27.8 ± 4.7 weeks of gestation, 31.5 ± 4.1 years of age, body mass index: 25.2 ± 11.3) participated in a randomized crossover design study. A flash glucose monitor and accelerometer were worn continuously for 7 days recording glycemic response, physical activity, and sleep. Nutritional intake and enjoyment of the exercise were self‐reported. Average heart rate during exercise was higher for HIIT (82 ± 4% HR_max_) compared with MICT (74 ± 4% HR_max_; *p* < 0.001) and participants achieved a peak heart rate of 92 ± 3% during HIIT (range 85%–97% HR_max_) compared with 81 ± 4% during MICT (*p* < 0.001). The change in glucose values from pre‐to‐postexercise were not different between conditions (HIIT: −0.62 ± 1.00 mmol/L; MICT: −0.81 ± 1.05 mmol/L; *p* = 0.300) with the exception that fewer individuals experienced postexercise hypoglycemia immediately following HIIT compared with MICT (8% versus 33% respectively; *p* = 0.041). Other glucose variables was not different between exercise protocols. Physical activity (*p* = 0.07) and caloric intake did not differ (*p* = 0.10). The majority of participants preferred HIIT (87.5%) and had greater perceived enjoyment compared to MICT (HIIT: 7.8 ± 1.5; MICT: 6.6 ± 2.0; *p* = 0.015). Sleep duration was 52 ± 73 min longer after participating in HIIT compared with the night prior (main effect for time *p* = 0.017); no significant changes for MICT. Overall, an acute session of HIIT appears to be well tolerated and demonstrates no adverse effects on maternal glycemic response.

## Graphical abstract

Study design to examine maternal glucose responses to an acute bout of HIIT versus MICT exercise.

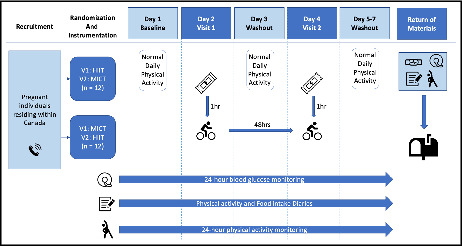

## 1 INTRODUCTION

Moderate‐intensity continuous training (MICT; i.e., 64%–76% of maximum heart rate; HR_max_) is the staple of prenatal physical activity research (ACOG Committee, 2020; Davies et al., 2019; Mottola et al., 2018). Guidelines from around the world recommend that pregnant individuals participate in 150 min of moderate‐intensity physical activity each week to achieve clinically meaningful health benefits (ACOG Committee, 2020; Davies et al., 2019; Mottola et al., 2018). The glucose‐lowering effects of moderate‐intensity prenatal exercise are well established and have been demonstrated to be an effective method in managing and preventing metabolic diseases (Davenport, Ruchat, et al., 2018; Davenport, Sobierajski, et al., 2018). Although the maternal glycemic response to vigorous (77–95% HR_max_) to near‐maximal intensities (≥96% HR_max_) remains essentially unknown, a dose–response reduction in maternal glucose has been identified with the rise in exercise intensity (Davenport, Sobierajski, et al., 2018). This has led to concerns of vigorous to near‐maximal exercise intensity causing hypoglycemia (<3.3 mmol/L; Seaquist et al., 2013). Hypoglycemia may lead to symptoms of cognitive dysfunction, poor coordination, and in severe cases, loss of consciousness which can increase the risk of trauma to the pregnant individual and fetus (Wowdzia & Davenport, 2021).

Due to metabolic adaptations occurring during pregnancy, pregnant individuals may be more prone to episodes of hypoglycemia compared with their nonpregnant state (Mazze et al., 2012). During pregnancy, an individual will experience a median decrease in maternal fasting plasma glucose of approximately 0.3 mmol/L compared with preconception levels (Riskin‐Mashiah et al., 2011). The decline in circulating glucose is multifactorial as a result of the increased metabolic costs of supporting the pregnancy including the maternal physiological adaptations (e.g., adipose tissue deposition, increased blood volume) and the addition of the fetal‐placental unit (Buschur et al., 2000; Riskin‐Mashiah et al., 2011). In order to counteract the progressive decline in maternal glucose, an individual's sensitivity to insulin decreases meaning they have a reduced ability to uptake circulating glucose from the blood into the skeletal muscle via insulin‐mediated pathways (Hunter & Garvey, 2004). However, noninsulin‐mediated glucose pathways via skeletal muscle contraction remain intact (Vargas et al., 2021). Thus it is important to investigate the glucose‐lowering effects of vigorous to near‐maximal exercise intensities during pregnancy.

An abundance of advice columns and prenatal workouts promoting vigorous‐intensity intervals continue to be developed in the absence of scientific evidence (Nagpal et al., 2021). In contrast, evidence‐based prenatal physical activity guidelines offer limited insight into the effects of interval training during pregnancy due to the paucity of available evidence. Yet, high‐intensity interval training (HIIT) has consistently ranked in the top seven fitness trends from 2014 to 2022 (Thompson, 2022) and consists of work intervals completed at intensities between 80% and 100% of peak heart rate interspersed with periods of lower intensity recovery or rest (Weston et al., 2014). In pregnant populations, HIIT has elicited greater levels of perceived enjoyment compared with MICT (Ong et al., 2016). Since enjoyment is an indicator of greater exercise adherence (Jekauc, 2015), HIIT may offer an additional method of increasing physical activity levels during pregnancy compared with MICT alone. In nonpregnant populations, HIIT has been demonstrated to be effective at increasing insulin sensitivity in the skeletal muscle for up to 48 h postexercise (Francois & Little, 2015). Over a period of 2 weeks, HIIT has demonstrated a 13% reduction in 24‐h blood glucose concentration and a 369% increase in GLUT4 protein content in individuals with type 2 diabetes (Little et al., 2011). These physiological adaptations increase the effectiveness of glucose reuptake by providing the skeletal muscle with a greater concentration of glucose transporters on the surface (Little et al., 2011; Richter & Hargreaves, 2013). Thus, HIIT has the potential to provide short‐ and long‐term health changes to fasting blood glucose (Little et al., 2011; Richter & Hargreaves, 2013). Despite the metabolic changes demonstrated in nonpregnant populations, the metabolic effects of HIIT have rarely been investigated during pregnancy. In 2016, Ong and colleagues compared 20 min of MICT (i.e., 65% HR_max_) to 21.5 min of HIIT (i.e., 6 × 15‐s self‐paced higher‐intensity work intervals [accumulation of 1.5 min of vigorous‐intensity exercise] interspersed with 3 min of moderate‐intensity cycling [i.e., 65% HR_max_]); Ong et al., 2016). Investigators reported the change in capillary glucose values from pre‐to‐postexercise were similar between conditions (MICT: 1.1 ± 0.2 mmol/L; HIIT: 1.0 ± 0.3 mmol/L) with no reports of postexercise hypoglycemia (Ong et al., 2016). However, this investigation remains limited in its ability to comment on HIITs effect on maternal glucose as traditional forms of HIIT (1:1 work to rest intervals, with longer durations of vigorous‐intensity exercise) were not examined. Given the dose–response relationship between maternal exercise intensity and decline in glucose, as well as lower hepatic glycogen stores during pregnancy (Davenport et al., 2016), vigorous to near‐maximal intensities obtained during aerobic HIIT (i.e. ≥90% HR_max_) may increase the risk of maternal postexercise hypoglycemia. Thus, the primary purpose of this study was to assess the feasibility of performing HIIT during pregnancy and the maternal glycemic response to an acute bout of HIIT compared with the traditional form of prenatal exercise, MICT. Fundamental aspects of physical performance, such as nutritional intake, sleep quality/duration, and physical activity (Charest & Grandner, 2020; Lee et al., 2017), will also be examined to assess the potential differences in exercise performance and identify any postintervention compensatory behaviors (e.g., increased sedentary time and caloric intake) which could undermine the health benefits achieved with MICT/HIIT (Pontzer et al., 2016).

## 2 HYPOTHESIS

We hypothesized that (a) maternal glucose would decrease from pre‐to‐postexercise in both the HIIT and MICT sessions; (b) the magnitude of the decrease in maternal glucose from pre‐to‐postexercise would be greater with HIIT compared with MICT; (c) 24‐ and 48‐h maternal fasting glucose would be lower for HIIT compared with MICT; and (d) the time spent hypoglycemic (<3.3 mmol/L) during the 24‐ and 48‐h periods would not be different between HIIT and MICT.

## 3 METHODS

### 3.1 Participants

Twenty‐four females (≥18 years of age, ≥20 weeks' gestation) carrying a singleton pregnancy were recruited between December 2020 and August 2021 to participate in this randomized crossover design study. Recruitment was open Canada‐wide and was done through convenience sampling. Participants had introductory phone calls to review informed consent and inclusion/exclusion criteria and to ask any questions prior to enrollment. Individuals were excluded if they had an absolute contraindication to physical activity during pregnancy as identified by the PARmed‐X for Pregnancy (Wolfe & Mottola, 2002). Individuals with relative contraindications were reviewed on an individual basis. Females were excluded if they had preexisting cardiovascular or respiratory disease, had multiple pregnancies (i.e., twins, triplets, or higher), or had a metabolic disease (e.g., type 1, type 2 diabetes, gestational diabetes mellitus). There was no fitness requirement or body mass index cutoff. Participants were given the option of completing the exercise visits in‐person in a private laboratory located at the University of Alberta or online via webcam. As a requirement of the study, all online participants were required to have access to a stationary bike, and sessions were booked around a secondary adult being present for additional safety. All exercise sessions were supervised by a certified clinical exercise physiologist. Approval for this study was received by the Health Research Ethics Board—Biomedical Panel of the University of Alberta (PRO‐00103630) and conformed to the guidelines outlined in the Declaration of Helsinki (Clinical Trials NCT05369247). The results presented in the current manuscript are part of a larger study examining the maternal and fetal physiological responses to HIIT and MICT exercise. Electronic signatures for informed consent were obtained from all participants prior to participation.

### 3.2 Study variables/instrumentation

#### 3.2.1 Demographics

Using a standardized intake form, demographics (i.e., age, height, weight, occupation, ethnicity, and health information concerning pregnancy status) were self‐reported by participants. The estimated delivery date was determined using the last menstrual period and later confirmed via ultrasound prior to participation.

#### 3.2.2 Anthropometrics

All participants were asked to measure and self‐report their height, current weight, and prepregnancy weight on the initial intake forms. Maternal weight was reported again during the first visit. In‐person participants had their measurements confirmed with a digital scale (400 Pound Physician Digital Scale; Angel, USA) and wall‐mount stadiometer.

#### 3.2.3 Glucose

At least 24 h prior to the first exercise session, a flash glucose sensor (Abbot Diabetes Care Inc.) was inserted onto the mid‐belly of the participant's left tricep. The flash glucose sensor was initialized by a handheld reader and required no further participant interaction, such as scanning the device, allowing for free‐living conditions and blinded subjects to their glucose values (Abbott Laboratories, 2021). Individuals wore the sensors for 7 days, allowing for 48‐h collection periods after each exercise test. Upon sensor removal, the sensor was scanned and uploaded offline. The daily pattern and glucose pattern reports were downloaded using the Freestyle Libre Pro Software for desktop (Abbott). Flash glucose sensors have been previously used with pregnant populations to assess glycemic profile and glycemic variation during the second and third trimesters of nondiabetic individuals (Nigam et al., 2020).

#### 3.2.4 Heart rate

Prior to the exercise session, participants were fitted with a continuous heart rate monitor (Online participants: POLAR H10 Heart Rate sensor; Polar Electro; In‐person participants: EQ‐LM100; Equivital Limited). Online participants downloaded the Polar Beat mobile application (downloaded through the App Store or Google Play) and logged in to the studies Polar.Flow account (flow.polar.com). Participants synced the heart rate monitors to the app which displayed their heart rate in beats per minute. Heart rate was then monitored throughout the exercise sessions by a certified clinical exercise physiologist (J.B.W.) and participants were coached to work within their prescribed heart rate zone.

#### 3.2.5 Interval timer

During the HIIT and MICT exercise session, an interval timer (www.intervaltimer.com) was displayed in front of the participants. The timer allowed for visual feedback regarding the length of intervals as well as the overall duration of the session (i.e., warm‐up, main bout, and cooldown).

#### 3.2.6 Rating of perceived exertion

Rating of percieved exertion (RPE) were reported after each work and active recovery interval during HIIT and every three‐min during the MICT session on the 6–20 Borg scale. At the end of each exercise session, participants were asked to consider their entire session (i.e., warm‐up, main bout, and cool down) and report their overall session RPE on the 6–20 Borg scale.

#### 3.2.7 Physical activity and sleep

Participants were also provided with an ActiGraph accelerometer (WGT3X‐BT; ActiGraph LLC). The ActiGraph was worn on a waist belt sitting on the right hip during waking hours and switched to a wrist strap before going to sleep. Participants kept a corresponding 7‐day journal that outlined wear‐time and any point at which the device was taken off for water‐based activities (e.g., showering). These journals also highlighted times at which participants went to sleep, woke up, or took naps throughout the day. Actigraph accelerometers have been previously validated in pregnant populations (Harrison et al., 2011).

#### 3.2.8 Nutritional intake

Participants kept a food diary for the entirety of the study (seven days) which detailed all meals, snacks, liquids, and nutritional supplements. The time of consumption was noted for each meal and snack. When possible, weighing or measuring the food was encouraged, and a visual aid was provided to help estimate food portion sizes. When cooking or baking, participants were asked to include recipes and any additional ingredient details such as brand names.

#### 3.2.9 Enjoyment

Following the competition of the exercise session, participants were asked to rank their overall enjoyment of the experience. Enjoyment was assessed on a 1–10 scale (1 hate, 3 unpleasurable, 5 neutral, 7 pleasant, 9 enjoyable). Once the participant had completed both MICT and HIIT sessions, they were asked to choose which session they ultimately preferred.

#### 3.2.10 Standardized snack

Participants were given two energy bars (i.e., Chocolate Chip Clif bars; Clif Bar & Company). Participants were instructed to consume one energy bar 1 h prior to their scheduled exercise appointments.

#### 3.2.11 Questionnaires

All questionnaires will be available via REDCap, a secure online website. Participants were asked to fill in a set of questionnaires: *Health History Questionnaire*, *Pittsburg Sleep Quality Index* (PSQI) which has been validated for use in pregnancy (Qiu et al., 2016), and a *Post‐partum and delivery Questionnaire* which inquiries about any complications that may have developed during their pregnancy.

### 3.3 Study design

Following enrollment into the study participants were randomized into two groups. The groupings decided which exercise protocol would be performed during visit 1, and the subsequent protocol for visit 2 (i.e., HIIT or MICT), thus removing the possibility of an order effect. Participants were then provided welcome packages that included a brief summary of the study, instructions on device application, and all materials required for participation. Participants were asked to fill in a set of questionnaires.

**FIGURE 1:**
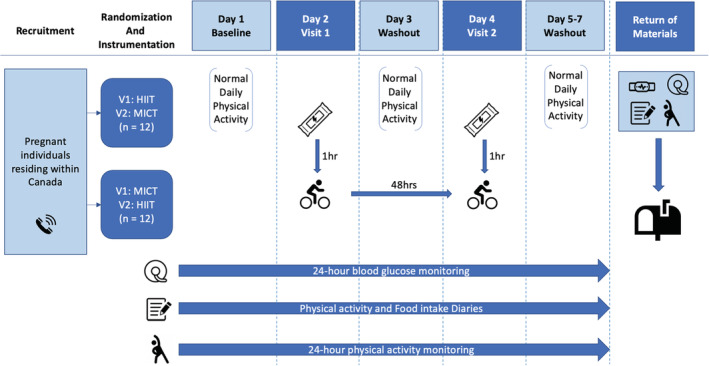
Study design. Abbreviation: HIIT, high‐intensity interval training; MICT, moderate‐intensity continuous training; Hr(s), hour(s); V1, visit one; V2, visit two. 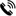: recruitment phone call; 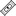: consumption of standardized snack; 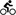: exercise intervention; 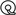: flash glucose monitor; 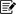: food and physical activity diaries; 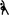: physical activity monitor; 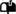: return of devices via mail.

Twenty‐four hours prior to the first exercise session, participants began wearing a flash glucose sensor, ActiGraph accelerometer, and tracking their daily food intake/physical activity/sleep logs. Twelve hours prior to each exercise session, participants were asked to refrain from caffeine, alcohol, and strenuous exercise. Participants consumed their standardized energy bar 1 h prior to their scheduled appointments. Participants would then engage in one acute bout of exercise per visit (i.e., one HIIT and one MICT session). Exercise visits were separated by at least 48 h. Acute exercise sessions were performed at approximately the same time of day for each individual. Participants continued to wear their physical activity monitors and complete their tracking sheets for at least 48 h after the competition of the second exercise protocol. The study design has been summarized in Figure 1.

### 3.4 Exercise intervention

**FIGURE 2:**
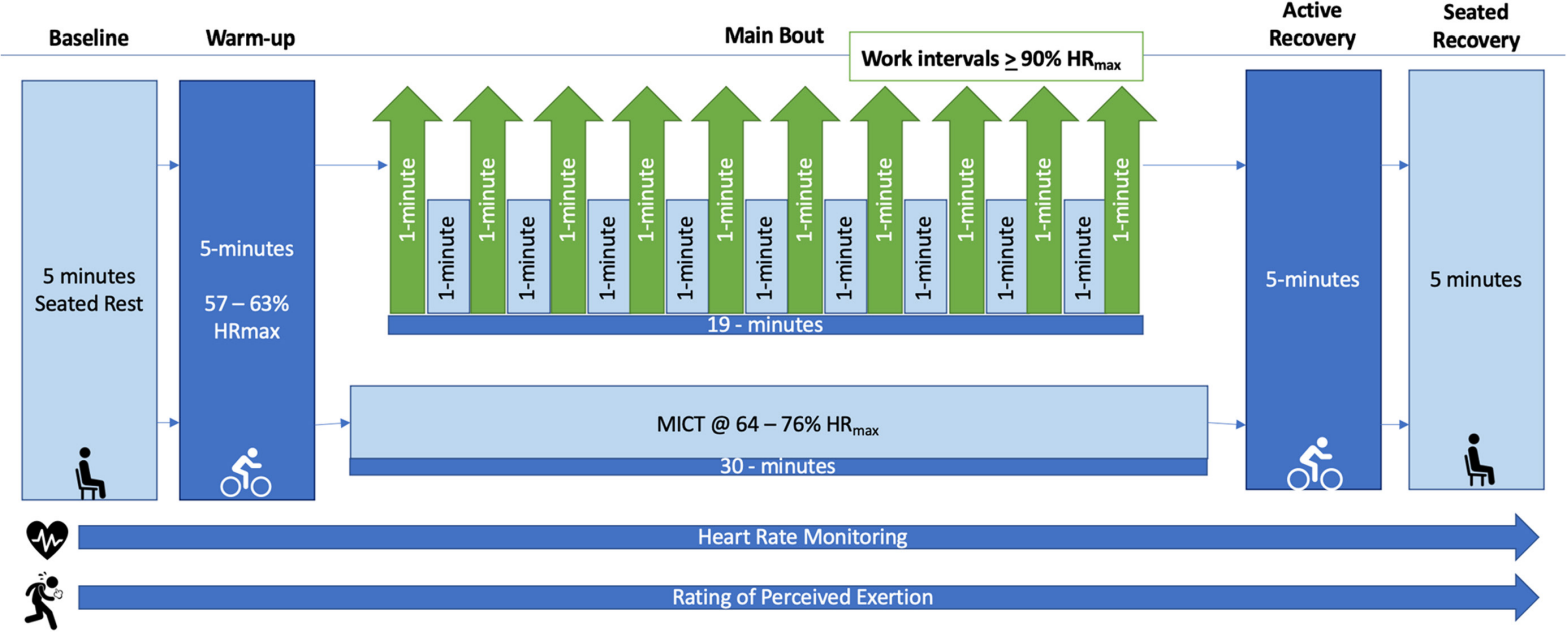
HIIT and MICT exercise intervention protocols. Abbreviations: HIIT, high‐intensity interval training; MICT, moderate‐intensity continuous training; HR_max_, maximal heart rate. 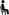: seated rest; 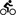: cycling on bike; 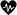: heart rate monitoring; 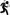: rating of perceived exertion.

The exercise protocols were not matched for energy expenditure or exercise volume, as they were based on two common/standard exercise protocols (Gillen et al., 2012; Little et al., 2011; Ram et al., 2021; Scott et al., 2019) to establish feasibility and generalizability. Each exercise session began with 5 min of quiet, seated rest followed by a 5 min warm‐up (57%–63% HR_max_) on a cycle ergometer. Participants then engaged in either the HIIT or MICT protocol, followed by a 5 min cooldown and finally 5 min of quiet seated rest. The HIIT protocol, modeled after previously published work (Gillen et al., 2012), consisted of 10 one‐min intervals of high‐intensity work (i.e., ≥90% HR_max_) interspersed with 9 one‐min intervals of self‐paced active recovery (19 min total). The MICT protocol consisted of 30 min of moderate‐intensity cycling (i.e., 64%–76% HR_max_). Exercise interventions are summarized in Figure 2.

### 3.5 Statistical analysis

#### 3.5.1 Sample size

The sample size was calculated based on a previous study examining the effect of light‐intensity versus vigorous‐intensity exercise on maternal pre‐to‐post‐exercise glucose in females at low risk for Gestational Diabetes Mellitus (GDM; Ruchat et al., 2012). Using G*Power 3.1 statistical power analysis program (Kiel, 2007), the total sample size was set at a desired power of 0.8, a cutoff for statistical significance of 0.05, and a calculated effect size of 0.8 (i.e., large). Based on this information the estimated sample size required 15 participants. Considering the risk of dropout, we aimed to recruit 24 participants.

#### 3.5.2 Statistical methods

Descriptive statistics were calculated as mean ± standard deviation and analysis occurred using statistical software (GraphPad PRISM 9 Software, San Diego, CA, USA). Paired parametric *t* tests were used to determine statistical differences between the HIIT and MICT protocols for maternal heart rate as well as the change in maternal glucose from pre‐to‐postexercise. Two‐way ANOVA was used, and the Holm‐Sidak post hoc test was performed to identify significant differences between groups for glucose (i.e., pre‐to‐postexercise, 24 and 48 h means, time spent hypo/hyperglycemic, and fasting glucose). In the event of missing data, a two‐way repeated measures mixed‐effects ANOVA was used. Wilcoxon matched‐pairs signed rank test was used for nonparametric data, including RPE and enjoyment. A Mann–Whiney test was used for unpaired data when comparing online versus in‐person rankings of perceived enjoyment for HIIT versus MICT. Fisher's exact test was used to determine the influence of prior engagement in HIIT on physical activity levels. McNemar's test for matched pairs was used to determine the difference in the rate of post‐exercise hypoglycemia between HIIT and MICT. Confidence intervals (95%) and effect sizes were reported where appropriate. Effect size can be categorized as small (0.2), medium (0.4), and large (0.8) (Krogh et al., 2015). Statistical significance was set at *p* < 0.05.

### 3.6 Data analysis

#### 3.6.1 Glucose outcomes

Interstitial glucose was used as a proxy for blood glucose and collected with the flash glucose monitor which was then downloaded with the FreeStyle Libre Software Version 1.0 software and imported to Microsoft Excel (Microsoft Software; Microsoft Corporation) to be analyzed. Data inspection included time‐matching glucose data to detailed food intake journals and exercise testing periods. Pre‐exercise glucose was taken just prior to warm‐up and compared with postexercise glucose values recorded immediately after the main bout. To determine the effect of exercise on mean interstitial fluid glucose concentrations, we analyzed 24 and 48 h periods in relation to the MICT and HIIT protocols. These time blocks began at the time point at which participants consumed their standardized pre‐exercise energy bar (i.e., 60 min prior to their scheduled assessments). Hypoglycemic trends were considered (i.e., percent time spent hypoglycemic) rather than the total number of glycemic events as per Abbott's recommendations (Abbort Laboratories, n.d.) and is in alignment with previous studies using continuous glucose monitors (Gillen et al., 2012). In consensus with the American Diabetes Association and the Endocrine Society Working Group, our cutoff for hypoglycemia in pregnant individuals was defined as glucose values <3.3 mmol/L, and hyperglycemia was defined as values ≥7.8 mmol/L (in alignment for 1 h postprandial target values; Seaquist et al., 2013).

For fasting glucose data, the values were recorded as glucose readings taken prior to the self‐reported awakening time verified by ActiGraph accelerometers. Three different mornings were taken into account per exercise protocol: the morning of the exercise testing day, the morning after the exercise protocol, and the subsequent morning. Participant values were averaged and contributed to the group mean and standard deviation.

#### 3.6.2 Maternal heart rate and rating of perceived exertion

Maternal heart rate was used to determine if participants were able to achieve the targeted heart rate zones set by each exercise protocol. Predetermined heart rate zones were calculated by using the age‐predicted equation [(220 − age) × desired intensity] for percent HR_max_. After the competition of an online participant session, heart rate data were exported from Polar.Flow (Polar electro) to Microsoft Excel (Microsoft Software; Microsoft Corporation). Exported heart rate data were then time matched with manually recorded heart rate values collected during the session. In‐person participant heart rate was continuously monitored by the same certified clinical exercise physiologist and recorded (LabChart and PowerLab; ADInstruments). All files were later cleaned for any irregulates (e.g., heart rate outliers caused by manually adjusting the belt) and exported into Microsoft Excel for further analysis.

Mean resting heart rate was determined over a 5‐min period during seated rest prior to starting exercise. Maximum/peak heart rate was determined as the highest point achieved during the main bout of the protocol and was translated into percent HR_max_. Average heart rate took into account the entirety of the main bout as outlined in Figure 2.

One participant's heart rate monitor failed to upload onto the Polar.Flow website after a HIIT session. Due to technological error, beat‐by‐beat analysis was not possible. However, during the session, the participant heart rate was recorded manually every 30–60 s during the protocol and therefore substituted for analysis.

RPE was reported throughout each of the MICT and HIIT sessions. Participants also reported one overall session RPE (i.e., considering warm‐up, the main bout, and the cooldown). Values were averaged and contributed to the group mean and standard deviations for each exercise protocol.

#### 3.6.3 Physical activity

Actigraph accelerometers were used to measure physical activity throughout the day. Accelerometers recorded accelerations over 60‐s time intervals (epoch) and were used to determine duration (summed duration of accelerations) and intensity (magnitude of accelerations) of movement throughout the day (Melanson & Freedson, 1995). Freedson bouts were used to determine the intensity of activity and broken down into sedentary (<100 counts per minute [cpm]), light (100–1951 cpm), and MVPA (≥1952 cpm) (Freedson et al., 1998). Accelerometers also reported wear time which was confirmed using self‐reported physical activity logs. Participant values were averaged and contributed to the group mean and standard deviations. Days with <600 min of wear time were excluded from the analysis. One participant did not wear the Actigraph accelerometer during the study and therefore their physical activity data could not be determined. The impact of HIIT/MICT on an individual's physical activity levels was assessed by considering four different time points per exercise visit: the day prior to the exercise test, the day of exercise protocol, and the following two days.

#### 3.6.4 Sleep outcomes

Self‐reported sleep logs determined sleep times in which a Cole‐Kripke algorithm was used to analyze the Actigraph data. Sleep data were broken down in five ways: total sleep time accumulated during the night, length of naps (i.e., occurred at least 45 min after reported awakening and lasted ≥20 min), combined total sleep time (i.e., sleep during the night and naps), time awake after sleep onset (WASO), and the number of awakening (i.e., subject woke up for a duration of 60 s or more after initial sleep onset; Fekedulegn et al., 2020). To compare HIIT and MICT, total sleep time accumulated during the night and quality of sleep indices were pulled from three different nights: the night leading up to the exercise test (i.e., prior to), the night of the exercise protocol, and the night immediately after completing the protocol.

#### 3.6.5 Nutritional intake

Participant's food journals were analyzed using ESHA Food Processor® Nutrition Analysis software version 11.9 for nutritional content including mean daily caloric (kcal), protein (g), fat (g), and carbohydrate (g) intake. The average daily intake was determined by analyzing all 7 days of the food diary. Furthermore, to determine the effect of caloric intake on our intervention, nutrition was analyzed over three time points: the standard 24 and 48 h glucose periods (i.e., beginning at the time point at which participants consumed their standardized snack [60 min prior to their scheduled assessments]) as well as 24 h prior to the first glucose period. Participants values were averaged and contributed to the group mean and standard deviations.

#### 3.6.6 Enjoyment

Participants' enjoyment was assessed using a 1–10 scale and overall preference for either HIIT or MICT was reported. Values were averaged and contributed to the group mean and standard deviations for each exercise protocol.

#### 3.6.7 Delivery and fetal outcomes

Data from the *Post‐partum and delivery Questionnaire* was used to determine pregnancy and fetal outcomes. Participant values for each outcome were averaged and contributed to the groups mean.

## 4 RESULTS

### 4.1 Participant demographics

Between December 2020 and August 2021, 24 pregnant females were recruited (27.8 ± 4.7 weeks gestation, range 21–37) and volunteered to participate in this randomized crossover design study. All individuals completed one HIIT and one MICT session. Six months prior to pregnancy, 83% of participants engaged in aerobic and/or resistance HIIT, and 71% continued after conception. Participant demographics are summarized in Table 1.

**TABLE 1:** Participant demographics

	Participants (*n* = 24)
**Age** (years)	31.5 ± 4.1
**Parity**	0.46 ± 0.78
**Ethnicity** , *n* (%)	
White/Caucasian	18 (75)
Asian/Pacific Islander	4 (16.7)
East Indian	1 (4.2)
First Nations, Metis, Inuit, or Alaska Native	1 (4.2)
**Prepregnancy body mass** (kg)	70.0 ± 11.3
**Prepregnancy BMI** , *n* (%)	25.2 ± 4.2
Underweight (<18.5)	0 (0)
Normal (18.5–24.9)	15 (62.5)
Overweight (25–29.9)	6 (25)
Obese (>30)	3 (12.5)
**Body mass at time of participation** (kg)	79.4 ± 11.6
**Participated in HIIT** , *n* (%)	
Within 6 months prior to pregnancy	20 (83.3)
Online (*n* = 12)	9 (75)
In‐person (*n* = 12)	11 (92)
After conception	17 (70.8)
Online (*n* = 12)	7 (58)
In‐person (*n* = 12)	10 (83)
**Maternal mass prior to delivery** (kg)	84.2 ± 11.8
**Pregnancy complications** (*n* = 21)	
Pregnancy‐related low‐back pain	12 (57)
Pelvic Girdle Pain	10 (47.6)
Urinary Incontinence	4 (19)
Preeclampsia	3 (14.3)
Gestational Hypertension	2 (9.5)
Prenatal Depression	1 (4.8)
Gestational Diabetes Mellitus	0 (0)
**Delivery outcomes** (*n* = 21)	
**Fetal birth mass** (g)	3423 ± 416
Microsomia (<2500 g); *n* (%)	1 (5)
Macrosomia (>4000 g); *n* (%)	3 (14)
**NICU;** *n* (%)	1 (4.8)

*Notes*: Unless otherwise indicated, values are expressed as mean ± standard deviation.

Abbreviations: %, percentage of total participants; BMI, body mass index; kg, kilograms; *n*, number of individuals; NICU, newborn intensive care unit; Parity, number of previous pregnancies that exceeded 20 weeks of gestation.

### 4.2 Acute exercise sessions

#### 4.2.1 Maternal heart rate and rating of perceived exertion

Pre‐exercise maternal heart rate was not different between the HIIT and MICT sessions; however, peak and mean heart rate, as well as RPE was higher during HIIT (see Table 2, *p* < 0.001; large effect size). All participants achieved heart rates ≥85% HR_max_ during the HIIT session with 18 individuals achieving intensities ≥90% HR_max_. Minor symptoms including transient lightheadedness (*n* = 2) and muscle cramps (*n* = 1) were reported during the acute HIIT session. One participant reported having pelvic girdle pain following their MICT session. No other adverse maternal or fetal outcomes were reported during or following the HIIT or MICT sessions.

**TABLE 2:** Maternal heart rate response and rating of perceived exertion to an acute HIIT and MICT session

	HIIT (*n* = 24)	MICT (*n* = 24)	*p*‐value	95% CI	Effect size
**Maternal heart rate**					
Resting HR (bpm)	84 ± 12	84 ± 12	0.730	−6.97, 6.97	0.00
Average HR During Exercise (bpm)	155 ± 8	140 ± 8	**<0.001**	10.35, 19.65	1.88
Peak HR achieved (bpm)	174 ± 7	152 ± 9	**<0.001**	17.32, 26.68	2.73
Peak HR Achieved (bpm)^a^	159–185	136–173	—		
**Relative intensity**					
Average HR during Main Bout (%HR_max_)	82 ± 4	74 ± 4	**<0.001**	5.68, 10.32	2.00
Average HR during WI (%HR_max_)	83 ± 4	—	—		
Average HR during RI (%HR_max_)	81 ± 4	—	—		
Peak HR achieved (%HR_max_)	92 ± 3	81 ± 4	**<0.001**	8.95, 13.05	3.11
Peak HR achieved (%HR_max_)^a^	85–97	71–88	—		
**Rating of perceived exertion**					
Average RPE	15 ± 1	12 ± 2	**<0.001**	2.08, 3.92	1.90
Max RPE Achieved	18 ± 1	13 ± 2	**<0.001**	4.08, 5.92	3.16
Overall session RPE^b^	16 ± 2	12 ± 2	**<0.001**	2.84, 5.16	2.00

*Notes*: Unless otherwise indicated, values are expressed as mean ± standard deviation. A paired parametric *t* test was used to determine statistical difference between groups for maternal heart rate. A Wilcoxon matched‐pairs signed rank test was used to determine statistical difference between groups for rating of perceived exertion. Significant values were bolded where appropriate.

Abbreviations: Bpm, beats per minute; CI, confidence interval; HIIT, high‐intensity interval training; HR, heart rate; %HR_max_, percentage of maximum heart rate achieved; MICT, moderate‐intensity continuous training; *n*, number of individuals; RI, recovery interval; RPE, rating of perceived exertion; WI, work interval.

^a^ Values are expressed as a range.

^b^ *n* = 22 for HIIT and MICT.

#### 4.2.2 Glucose

Interstitial glucose values were not different between HIIT or MICT pre‐ or postexercise (*p* = 0.510). Other metrics of maternal glycemic response, including 24‐h and 48‐h (*p* = 0.169) recovery, time spent <3.3 mmol/L (*p* = 0.141) and ≥7.8 mmol/L (*p* = 0.197), as well as fasting glucose (*p* = 0.865), were not different between conditions. Immediately after HIIT, fewer individuals experienced postexercise hypoglycemia (<3.3 mmol/L) compared with MICT utilizing flash glucose monitoring (see Table 3), the small effect size for all measures.

**TABLE 3:** Glucose in response to acute HIIT and MICT

	HIIT (*n* = 24)	MICT (*n* = 24)	*p*‐value (Interaction)	*p*‐value (Exercise)	*p*‐value (Time)	95% CI	Effect size
**Acute exercise**			0.510	0.755	**<0.0001**		
Pre‐exercise glucose (mmol/L)	4.76 ± 0.98	4.80 ± 0.88				−0.58, 0.50	−0.04
Postexercise glucose (mmol/L)	4.15 ± 0.62	3.98 ± 0.98				−0.31, 0.65	0.21
Change in glucose pre‐to‐postexercise (mmol/L)^b^	−0.62 ± 1.00	−0.81 ± 1.05	0.300			−0.41, 0.79	0.19
Postexercise hypoglycemia; *n* (%)^a^	2 (8)	8 (33)	**0.040**				
Online participants (*n*)	2	5					
In‐person participants (*n*)	0	3					
**Recovery**			0.169	0.896	0.388		
Mean 24‐h glucose (mmol/L)	4.30 ± 0.44	4.34 ± 0.43				−0.29, 0.21	−0.09
Mean 48‐h glucose (mmol/L)	4.38 ± 0.40	4.35 ± 0.4				−0.20, 0.26	0.08
**Time spent < 3.3 mmol/L**			0.141	0.960	0.111		
24 h (%)	10.37 ± 13.50	8.81 ± 11.79				−5.80, 8.92	0.12
48 h (%)	6.97 ± 1.32	7.25 ± 9.29				−4,14, 3.58	−0.04
**Time spent ≥ 7.8 mmol/L**			0.197	0.238	0.211		
24 h (%)	0.22 ± 0.53	0.35 ± 0.96				−0.58, 0.32	−0.17
48 h (%)	0.00 ± 0.00	0.36 ± 1.32				−0.9, 0.18	−0.39
**Fasting glucose**			0.865	0.737	0.1533		
Pre‐exercise morning (mmol/L)	3.55 ± 0.80	3.53 ± 0.65				−0.40, 0.44	0.03
Postexercise morning 1 (mmol/L)	3.70 ± 0.57	3.70 ± 0.63				−0.35, 0.35	0.00
Postexercise morning 2 (mmol/L)	3.75 ± 0.62	3.70 ± 0.52				−0.28, 0.38	0.09

*Notes*: Data retrieved from flash glucose monitor. All values are expressed as mean ± standard deviation. A two‐way repeated measures ANOVA was used to determine statistical difference between groups unless otherwise indicated. A McNemar's test (indicated by ^a^) or paired parametric t test (indicated by ^b^) were also used to determine statistical difference between groups. Significant values were bolded where appropriate.

Abbreviations: CI, confidence interval; HIIT, high‐intensity interval training; MICT, moderate‐intensity continuous training; *n* (%), number of individuals and percentage of total participants.

#### 4.2.3 Rating of perceived enjoyment

Participants (*n* = 24) reported greater overall enjoyment of HIIT compared with MICT (7.8 ± 1.51 versus 6.6 ± 1.98, respectively. *p* = 0.015) with 87.5% reported preferring HIIT. Perceived enjoyment of HIIT between online and in‐person participants did not differ (7.6 ± 1.56 versus 7.9 ± 1.51, respectively, *p* = 0.655) nor did MICT (5.9 ± 1.83 versus 7.25 ± 1.96, respectively 0.0887).

#### 4.2.4 Physical activity, nutrition, and sleep

Overall, physical activity and nutritional intake were not different between MICT and HIIT conditions. Of the 22 participants who wore their Actigraph accelerometers, 16 individuals met the 150 min of moderate‐intensity physical activity per week prescribed by the 2019 Canadian Guidelines for Physical Activity throughout pregnancy (Mottola et al., 2018). Participants who had previously engaged in HIIT prior to or during their current pregnancy had a greater likelihood of achieving 150 min of moderate‐intensity physical activity each week (*p* = 0.046 and 0.004, respectively) compared to those with no HIIT experience. Physical activity and nutrition are summarized in Table 4.

**TABLE 4:** Physical activity intensity prior to and following exercise intervention

Physical activity	HIIT (*n* = 22)				MICT (*n* = 22)				*p*‐value (Exercise)	*p*‐value (Time)	*p*‐value (Interaction)
	*Day Prior*	*Day of*	*Post‐1*	*Post‐2*	*Day Prior*	*Day of*	*Post‐1*	*Post‐2*			
Sedentary (min/day)	578 ± 165	611 ± 41	617 ± 166	596 ± 23	552 ± 68	610 ± 29	569 ± 157	548 ± 167	0.130	0.100	0.450
Light (min/day)	263 ± 102	251 ± 68	232 ± 64	263 ± 99	241 ± 63	260 ± 68	270 ± 96	275 ± 85	0.450	0.320	0.070
MVPA (min/day)	27 ± 20	21 ± 15	26 ± 24	25 ± 23	31 ± 6	31 ± 26	34 ± 30	21 ± 14	0.260	0.270	0.360

Nutrition^a^	*Prior*	*24 h*	*48 h*	*Prior*	*24 h*	*48 h*			
Calories (kcal)	2596 ± 876	2745 ± 1023	2772 ± 795	2441 ± 759	2448 ± 733	2575 ± 666	0.100	0.630	0.900
Protein (g)	112 ± 7	109 ± 40	112 ± 45	100 ± 39	100 ± 39	102 ± 38	0.110	0.920	0.990
Carbs (g)	316 ± 120	338 ± 133	323 ± 113	314 ± 128	300 ± 71	308 ± 63	0.310	0.980	0.710
Fat (g)	101 ± 45	114 ± 71	119 ± 52	96 ± 40	100 ± 47	107 ± 39	0.170	0.320	0.910

Sleep^b^	*Prior to*	*Night of*	*Following*	*Prior to*	*Night of*	*Following*			
At night (min)	398 ± 70	451 ± 68	412 ± 84	419 ± 66	431 ± 61	422 ± 77	0.842	**0.017**	0.157
Naps (min)	1 ± 7	11 ± 29	28 ± 44	3 ± 11	16 ± 38	6 ± 14	0.321	**0.038**	0.052
CS (min)	400 ± 68	462 ± 64	440 ± 88	422 ± 64	446 ± 61	428 ± 75	0.779	**<0.001**	0.144
WASO (min)	66 ± 35	72 ± 41	61 ± 45	60 ± 37	58 ± 38	72 ± 42	0.473	0.762	**0.032**
Awakenings^c^	17 ± 9	19 ± 11	14 ± 8	16 ± 8	15 ± 8	17 ± 10	0.337	0.684	**0.012**

*Notes*: Data derived from Actigraph accelerometer and daily food logs. All values are expressed as mean ± standard deviation. Two‐way repeated measures ANOVA mixed effect was used to determine statistical difference between groups.

Abbreviations: Carbs, carbohydrates; CS, combined sleep (i.e., sleep at night and naps); g, grams; HIIT, high‐intensity interval training; h, hours; kcal, kilocalories; MICT, moderate‐intensity continuous training; Min, minutes; MVPA, moderate‐to‐vigorous physical activity; *n*, number of participants; WASO, wake after sleep onset.

The bolded values are to highlight the significan values.

^a^ *n* = 24 for nutrition.

^b^ *n* = 23 for sleep metrics.

^c^ Number of awakenings after onset of sleep.

The Pittsburg Sleep Quality Index revealed 70% of participants reported being as “bad” sleepers with an average duration of 7 ± 0.2 h per night. As summarized in Table 4, participants experienced longer sleep durations after participating in HIIT (main effect for time points: *p* = 0.017). Mixed effect‐multiple comparisons analysis further identified a significant difference between sleep duration prior to participation (*p* = 0.003) and the day following participation (*p* = 0.028) in comparison to sleep duration immediately after HIIT participation. No effect for time points was observed for the MICT protocol. A post hoc mixed effect analysis also indicated a main effect for time points (*p* = 0.019), demonstrating a greater number of awakenings after participating in HIIT (19 ± 11) compared with the night after HIIT participation (14 ± 8).

#### 4.2.5 Postpartum and delivery Questionnaire

Three participants were lost to follow up for the postpartum questionnaire inquiring about pregnancy/delivery and fetal outcomes. Babies were born at term (39 ± 1.2 weeks gestation), primarily by vaginal delivery (*n* = 12; 57%). Only one admission to the newborn intensive care unit (NICU) was reported. All other pregnancies, labor, and delivery outcomes are summarized in Table 1.

## 5 DISCUSSION

The present study compared the effects of an acute bout of HIIT versus MICT on maternal glucose concentrations in 24 pregnant participants. Overall, we demonstrated no difference in the glucose response to an acute bout of exercise (i.e., change in glucose from pre‐to‐postexercise as well as the 24‐ and 48‐h responses) between conditions with the exception that fewer participants experienced postexercise hypoglycemia following HIIT compared with MICT, whereas previous HIIT studies have demonstrated peak maternal heart rates between 80% and 90% HR_max_ (Anderson et al., 2021; Ong et al., 2016), 75% of our participants were able to achieve the target heart rate goal of ≥90% maternal HR_max_ with the highest achieved maternal heart rate equating to 97% HR_max_. Physical activity and caloric intake did not differ between days or conditions; however, participants experienced prolonged sleep duration after participating in HIIT along with an increased number of nightly awaking. Finally, despite greater ratings of perceived exertion from the HIIT session, participants in our study indicated that they experienced higher levels of perceived enjoyment during HIIT compared with MICT. Our findings contribute important insights into the impact of acute HIIT on maternal glycemic response and provide evidence of aerobic HIIT being well‐tolerated during pregnancy.

### 5.1 Postexercise hypoglycemia

The glucose‐lowering effects of maternal exercise are well established. Data from a recent systematic review and meta‐analysis demonstrate an average 0.6 mmol/L reduction in maternal capillary glucose in response to an acute bout of light‐to‐vigorous intensity exercise with relatively low risk (0%–4%) of inducing hypoglycemia (Davenport, Sobierajski, et al., 2018). The meta‐regression analysis also indicated a dose–response relationship between exercise intensity/duration and maternal glucose utilization but very limited information on vigorous to near‐maximal intensities (Davenport, Sobierajski, et al., 2018). In the current study, the standardized exercise sessions were based on commonly used MICT and HIIT protocols. (Gillen et al., 2012; Ram et al., 2021). However, the exercise protocols were not matched for the volume of physical activity as it was greater for MICT (i.e., 64%–76% HR_max_ for 30 min; 240 min per session [MET]) compared with HIIT (i.e., 10 min of ≥90% HR_max_ with 9 min of self‐paced recovery; 153 MET). Assuming a dose–response relationship, the greater volume of physical activity would account for a larger decline in maternal glucose and increased rates of postexercise hypoglycemia demonstrated within our MICT condition compared with HIIT.

Flash glucose monitors provide a greater understanding of changes in glucose values with continuous readings reported in 15‐min averages compared with conventional fingerstick blood samples which are traditionally only taken pre‐ and postexercise. It has been observed that interstitial glucose values remain within the close approximation of blood glucose concentrations (i.e., mean difference of 0.13 ± 0.03) with the exception of when systemic blood glucose decreases <3.3 mmol/L (Caplin et al., 2003). This is common among commercially available glucose monitors with the lowest sensor accuracy occurring during hypoglycemia and the highest accuracy during hyperglycemia (Aberer et al., 2017). During periods of hypoglycemia, interstitial glucose values decline more rapidly and reveal lower concentrations compared with capillary blood glucose (Caplin et al., 2003). Despite up to 33% of participants experiencing postexercise hypoglycemia after MICT and 8% after HIIT, they were not symptomatic (e.g., experiencing dizziness or lightheadedness). Overall, it would appear that an acute session of HIIT does not appear to increase the odds of postexercise hypoglycemia in comparison with MICT.

### 5.2 Fasting glucose

Fasting glucose can serve as an indication of the effects of prenatal exercise on maternal glycemic control (Davenport, Sobierajski, et al., 2018). It is expected that the glucose‐lowering effects of vigorous to near‐maximal exercise intensity obtained during HIIT may increase insulin sensitivity up to 48 h postintervention (Francois & Little, 2015). In combination with lower maternal hepatic glycogen stores (Davenport et al., 2016), fasting glucose may be acutely reduced during pregnancy. As observed in nonpregnant normoglycemic populations, HIIT has been effective at lowering fasting glucose values (i.e., reduction of 0.13 mmol/L compared with baseline) following an intervention lasting at least 2 weeks (Jelleyman et al., 2015). Contrary to these findings, our study demonstrated that neither HIIT nor MICT altered maternal fasting glucose on the two mornings following participation. This may be due to the physiological adaptations of a normoglycemic pregnancy, including an increased rate of gluconeogenesis in the liver and increased number/size of beta cells (Lain & Catalano, 2007). In support of this, meta‐analyzed data have demonstrated that nondiabetic normoglycemic pregnant individuals have demonstrated no change in fasting glucose after chronic participation in light‐to‐vigorous‐intensity physical activity (Davenport, Sobierajski, et al., 2018). Thus, it is reasonable to suggest that an acute HIIT session does not adversely affect fasting glucose during pregnancy in comparison with MICT.

In the present study, the average maternal fasting glucose prior to and following MICT and HIIT exercise sessions ranged from 3.50 to 3.75 mmol/L. This is slightly lower than expected, as previous reports of maternal fasting glucose values are anticipated to be ~4.2 mmol/L within the second and third trimester of pregnancy (Riskin‐Mashiah et al., 2011). The discrepancy of lower maternal glucose values may be a result of sensor error and/or the blood‐interstitial fluid glucose concentration gradient. However, our findings may also suggest that pregnant individuals may be prone to experiencing asymptomatic nocturnal hypoglycemia as this has been previously reported in pregnant populations with type 1 and type 2 diabetes, suggesting similar time spent below 3.9 and 2.8 mmol/L (i.e., 1.3–1.5 and 0.3–0.5 h, respectively; Murphy et al., 2007). An alternative explanation for lower than expected maternal fasting glucose values may be due to the placement of the flash glucose sensors. When flash glucose sensors are compressed, such as when they are being slept on overnight, interstitial fluid volume is reduced and the sensor's sensitivity to glucose decreases (Gibb et al., 2020). Thus, future investigations would benefit from fingerstick blood samples to confirm nocturnal glycemic trends.

### 5.3 24‐ and 48‐h glucose

Contrary to our hypothesis, 24‐ and 48‐h mean maternal glucose was not different between the MICT and HIIT protocols. Although our HIIT protocol previously demonstrated 24‐h reductions in mean glucose in nonpregnant‐type two diabetics (Gillen et al., 2012), it is possible that greater volumes of HIIT are needed to observe an effect in nondiabetic normoglycemic individuals. As expected, time spent in hyperglycemia (i.e., ≥7.8 mmol/L) was similar after participating in HIIT and MICT. Although no signs or symptoms of hypoglycemia were reported after participation in HIIT and MICT, our participants spent approximately 2‐to‐2.5‐h <3.3 mmol/L throughout the day and overnight within the 24‐h period. These values are not unexpected as similar percent time spent hypoglycemic (i.e., 2 h) has been reported in asymptomatic normoglycemic pregnant individuals in the absence of an exercise intervention (Porter et al., 2004). However, further research utilizing capillary or blood glucose values as well as changes in insulin sensitivity to confirm these findings is warranted.

### 5.4 Enjoyment

Time efficiency is one of the leading appeals of HIIT as it overcomes the primary barrier to physical activity (i.e., perceived lack of time) requiring ~40% less time commitment to achieve similar health benefits to MICT (Wewege et al., 2017). This may be appealing to expecting mothers as current adherence rates to the physical activity guidelines (i.e., 150 min of moderate‐intensity physical activity per week; Mottola et al., 2018) are low (i.e., ~15%; Evenson & Wen, 2011). It is well accepted that perceived enjoyment can be a psychological motivator for increasing physical activity adherence (Jekauc, 2015) as well as an indicator of future participation (Ryan et al., 1997). It is important to acknowledge the potential for self‐selection bias within the present findings considering the high percentage of participants with prior HIIT experience and the voluntary nature of the study. However, the finding that pregnant individuals had greater enjoyment of HIIT compared with MICT in our study and others warrants further investigation. (Ong et al., 2016)

### 5.5 Physical activity and caloric intake

Traditionally, the strenuous nature of vigorous‐intensity exercise has led to concerns that the benefits of these activities, such as HIIT, would be counterbalanced by a reduction in overall physical activity levels outside of the exercise session due to an increase in perceived fatigue (Pontzer et al., 2016). Through objective measures of free‐living physical activity and self‐reported dietary records, we were able to examine whether an acute bout of HIIT during pregnancy resulted in compensatory behaviors. Compensatory behaviors are concerning as they may undermine the health benefits achieved through physical activity (Pontzer et al., 2016). Despite the significantly greater session and exercise rating of perceived exertion during HIIT, objectively monitored physical activity patterns (i.e., sedentary, light, and moderate‐to‐vigorous intensity physical activity) were not different on the day prior to, of, or following HIIT compared with MICT. Although self‐reported dietary recall has demonstrated significant limitations (Dhurandhar et al., 2015), particularly with pregnant individuals underestimating their daily energy intake by 45% (McGowan & McAuliffe, 2012), our crossover study design demonstrated no difference in energy intake (i.e., total calories, protein, fat, or carbohydrates) between days or MICT and HIIT conditions. Similar findings have been found in older adults and individuals undergoing cardiac rehab in which HIIT did not result in reduced daily energy expenditure (Bruseghini et al., 2020), increased sedentary minutes (Bruseghini et al., 2020), or dietary compensations (Taylor et al., 2021). Thus, our findings support that an acute session of aerobic HIIT does not appear to facilitate adverse lifestyle modifications during pregnancy.

### 5.6 Sleep

Sleep is an essential process that aids in normal physiological functioning during pregnancy. The National Sleep Foundation recommends that adults (18–60 years old) should sleep at least 7 h per night on a regular basis and approximately 9 h when recovering from sleep debt or illness (Watson et al., 2015). In the current study, participants narrowly satisfied sleep recommendations with only 6.6–7 h of sleep per night. It is established up to 97% of pregnant females report disturbed sleep by the third trimester (Da Silva et al., 2005; Mindell et al., 2015) with commonly reported reasons including physical discomforts, fetal movement, acid reflux, and increased frequency of urination (Mindell et al., 2015). A longitudinal study reported that as pregnancy progresses, individuals experience shorter durations of sleep at night (i.e., decreasing from 7.61 to 6.85 h per night) as well as an increase in the number and overall duration of awakenings (Mindell et al., 2015). Short sleep duration (<6 h per night) has been associated with adverse pregnancy outcomes including prolonged labor (Lee & Gay, 2004), preterm delivery (Kajeepeta et al., 2014), and a 450% increase in the odds of having a cesarean delivery (Lee & Gay, 2004). Optimizing sleep is critical to maternal/fetal health. Previous studies have established that low‐to‐moderate intensity physical activity improves sleep duration and quality in pregnant populations (Ozkan & Rathfisch, 2018; Tan et al., 2020). In nonpregnant populations, aerobic HIIT interventions (i.e., 12 weeks) saw improved sleep quality and decreased fatigue (Jiménez‐García et al., 2021). Similar to previous findings, after engaging in HIIT, our participants demonstrated an increase in sleep duration to ~7.5 h (i.e., 52 ± 73 min longer compared with the night prior), whereas no significant change was seen with MICT (i.e., 12 ± 63 min longer compared with the night prior). The clinical significance of this finding is unclear, while the novelty of the training stimulus may have contributed to longer sleep duration, participants also experienced the highest number of awakenings after participating in HIIT. Further work is required to better understand the impact of HIIT on maternal sleep patterns.

### 5.7 Strengths and limitations

Strengths of the current study include its randomized crossover design removing participant variability between interventions and its large sample size for data analysis. The chosen exercise protocols were effective in eliciting targeting heart rates as well as achieving a glycemic response from participants. Our findings also demonstrate high external validity as 50% of participants were able to participate from their homes thus demonstrating the feasibility of how aerobic HIIT can be implemented in a number of different settings. It is also important to acknowledge that no participant in our study reported developing GDM during their pregnancy. Thus, our sample did not have any metabolic complications that may have influenced the glucose values.

A limitation of our study was that it refrained from testing females before the 20th week of gestation therefore the results are limited in their ability to address maternal response to HIIT throughout the entirety of the pregnancy. In addition, a single bout of HIIT is unable to predict the cumulative effect of multiple sessions on maternal well‐being. Future studies may also benefit from prepackaged meals to better standardize nutritional intake between exercise trials. The flash glucose monitors utilized in the present study have demonstrated low accuracy in reporting glucose values when <3.3 mmol/L, therefore, our hypoglycemic values should be interpreted with caution (Abbort Laboratories, n.d.). Device malfunction and user error (e.g., forgetting to apply accelerometer or battery failure, glucose sensor falling off in two participants resulting in <48‐h collection after the second visit—i.e., one HIIT and one MICT session) also contributed to limitations of the data, thus a mix‐effect analysis was necessary. Finally, the enjoyment scale utilized in the present study has not been validated for use in pregnancy.

## 6 CONCLUSION

An acute bout of aerobic HIIT demonstrates no adverse effects on maternal glycemic response over a 48‐h period and appears to be well‐tolerated during pregnancy. HIIT also appears to generate similar maternal glycemic responses in comparison to MICT. Further research is needed to identify the safety and potential benefits of HIIT, as the highly enjoyable activity continues to be performed by many individuals after conception.

## AUTHOR CONTRIBUTIONS

The listed authors conceived the study together. Jenna B. Wowdzia did data collection and analysis. All authors contributed to the interpretation and revision process.
